# Histological Features and Biocompatibility of Bone and Soft Tissue Substitutes in the Atrophic Alveolar Ridge Reconstruction

**DOI:** 10.1155/2016/3608602

**Published:** 2016-02-23

**Authors:** Carlo Maiorana, Mario Beretta, Davide Rancitelli, Giovanni Battista Grossi, Marco Cicciù, Alan Scott Herford

**Affiliations:** ^1^Center for Edentulism and Jawbone Atrophies, Fondazione IRCCS Policlinico, Department of Oral Sciences, University of Milan, 20141 Milan, Italy; ^2^Oral Surgery and Implantology, Fondazione IRCCS Policlinico, University of Milan, 20141 Milan, Italy; ^3^Human Pathology Department, School of Dentistry University of Messina, 98100 Messina, Italy; ^4^Oral and Maxillofacial Surgery, Loma Linda University School of Dentistry, Loma Linda, CA 95231, USA

## Abstract

The reconstruction of the atrophic alveolar ridges for implant placement is today a common procedure in dentistry daily practice. The surgical reconstruction provides for the optimization of the supporting bone for the implants and a restoration of the amount of keratinized gingiva for esthetic and functional reasons. In the past, tissue regeneration has been performed with autogenous bone and free gingival or connective tissue grafts. Nowadays, bone substitutes and specific collagen matrix allow for a complete restoration of the atrophic ridge without invasive harvesting procedures. A maxillary reconstruction of an atrophic ridge by means of tissue substitutes and its histological features are then presented.

## 1. Introduction

Prosthetically guided implantology provides for the creation of an optimal bone support to dental implants in order to guarantee an adequate prosthetic restoration. Moreover, the reconstruction of the soft tissues around the implant is essential, in order to reduce the risk of peri-implant mucositis and peri-implantitis [1, 2]. Such sophisticated and multiple stepped treatments include three to four surgical phases usually from 12 to 18 months to be concluded. With the purpose of reducing the invasiveness of the treatment, bone substitutes have been used within the last 20 years to avoid autogenous bone harvesting [3, 4]. In the last five years, even soft tissue substitutes have been tested to avoid the use of autogenous free gingival or connective tissue grafts [5, 6]. The association of bone regeneration techniques, soft tissues reconstruction, and devices able to maintain the biomaterials in site for at least six to eight months prior to implant placement is nowadays a widely documented approach. The purpose of this paper was to show a paradigmatic clinical case of an upper jaw alveolar atrophy, in which a titanium mesh, xenogeneic bone, and a collagen porcine matrix were used to restore the anatomic integrity of the deficient ridge as well as the soft tissues around the implants.

## 2. Materials and Methods

A 56-years-old. female patient, in good general health status, was examined at the Department of Implantology, Fondazione IRCCS Cà Granda, Ospedale Maggiore Policlinico, Milan, Italy. The patient was asking for the rehabilitation of the upper left edentulous maxilla with a fixed implant-supported prosthesis. The clinical appearance of the edentulous ridge showed a vertical and horizontal contraction with a cross bite intermaxillary relationship. On the right side, an extended sinus could be appreciated in the edentulous posterior area. The radiographic examination, carried out by means of a panoramic radiograph and a CBCT scan, confirmed the atrophy, characterized by a narrow knife-edge ridge in the bicuspid area and a residual ridge 3 mm in height associated with an expanded maxillary sinus in the molar area. Patient undersigned an informed consent and the treatment was performed accordingly with the declaration of Helsinki rules.

The rehabilitation plan consisted in a three-step surgical treatment:

- bone reconstruction of the atrophic ridge with autogenous chips, xenogeneic bone, titanium mesh, and bilateral sinus elevation with xenogeneic bone,
- titanium mesh removal after seven months and soft tissues reconstruction with a collagen matrix,
- implant placement,
- prosthetic rehabilitation with acrylic temporary prosthesis followed by a gold ceramic bridge.

Firstly the patient underwent a full mouth disinfection session, and proper home oral hygiene instructions were given. Sinus was prepared 15 days prior to surgery with mometasone nasal spray (Nasonex® Merck, Sharp and Dhome, Ch), twice a day. During the first surgical session, under general anesthesia, a full thickness flap from upper left canine to the maxillary tuberosity was reflected, a sinus elevation procedure with a lateral approach was conducted, and the subantral cavity was filled with xenogeneic bone (Bio-Oss, Geistlich®, Wohlusen, Ch). The ridge reconstruction was performed with a 1 : 1 ratio mixture of autogenous bone harvested with a bonescraper from the tuberosity and xenogeneic bone particles. The mix was maintained in place with a titanium mesh (OMNIA S.p.A., Fidenza (Parma), Italy) screwed to the recipient site with bone screws. A collagen resorbable membrane (BioGide, Geistlich, Wolhusen, Chiasso) was adapted over the mesh in order to limit the soft tissue cells migration in the grafted area and to promote soft tissues creeping in case of wound dehiscence and early exposure of the mesh.

Rehrmann periosteal incisions were performed at the buccal flap and a two-layer suture with a 4-0 polyglactin (Vycril®, Ethicon, USA) wire was done to seal the tissues. On the right side, a lateral approach sinus elevation was done at the first molar site and the regeneration was done with xenogenic bone. Sutures were removed after 15 days. The healing preceded uneventfully and after seven months the second surgery for mesh removal and soft tissues reconstruction was carried out. Under local anesthesia, the titanium mesh was exposed to a midcrestal incision and then removed. After the titanium mesh removal a sample of the regenerated tissue was taken by means of a trephine bur for the histological evaluation.

The buccal flap was apically repositioned to the underlying connective tissue to recreate the vestibule and the exposed tissue was covered with a porcine collagen matrix (Mucograft®, Geistlich, Wolhusen, Chiasso), which was secured to the recipient site with 5-0 polyglactin sutures. Forty days after the surgery, a complete healing of the soft tissue was observed and the third step for implant placement was executed. A full thickness flap was elevated both at the right and left sites and five implants (Camlog Screw line®, Camlog AG, Ch) were placed. After implant placement in the prosthetically driven position, a small horizontal bony defect was present at the canine level and the correction was done by means of Bio-Oss and Biogide®.

**Figure 1:**
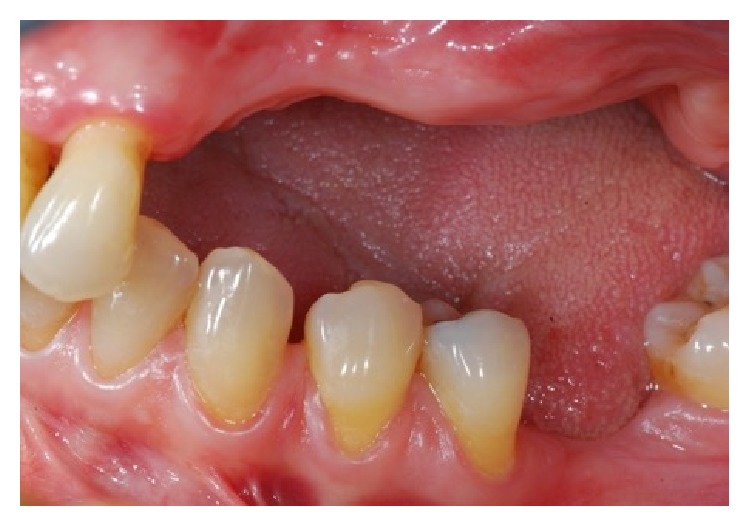
Clinical view of the left upper maxilla atrophic ridge.

**Figure 2 fig2:**
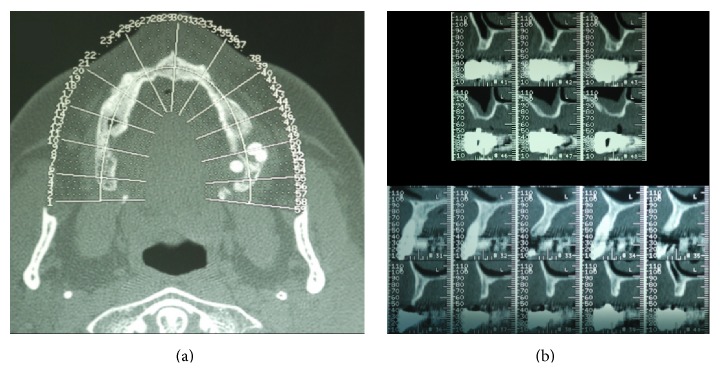
The CT dental scan confirms the clinical view showing atrophic maxillary ridge with important horizontal bone defect. (a) It is possible to underline all the atrophies of the upper maxilla; (b) a particular of the axial section of the area involved in the grafting procedures.

**Figure 3 fig3:**
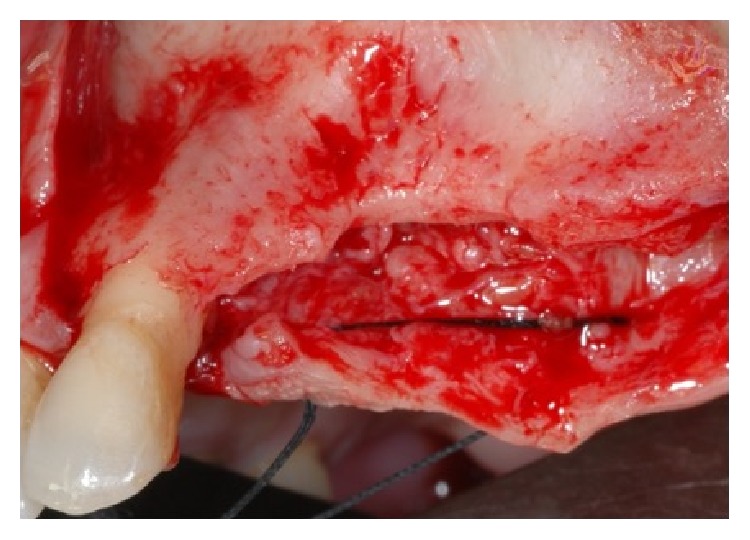
Clinical view of the bone after the elevation of the mucoperiosteal flap.

**Figure 4 fig4:**
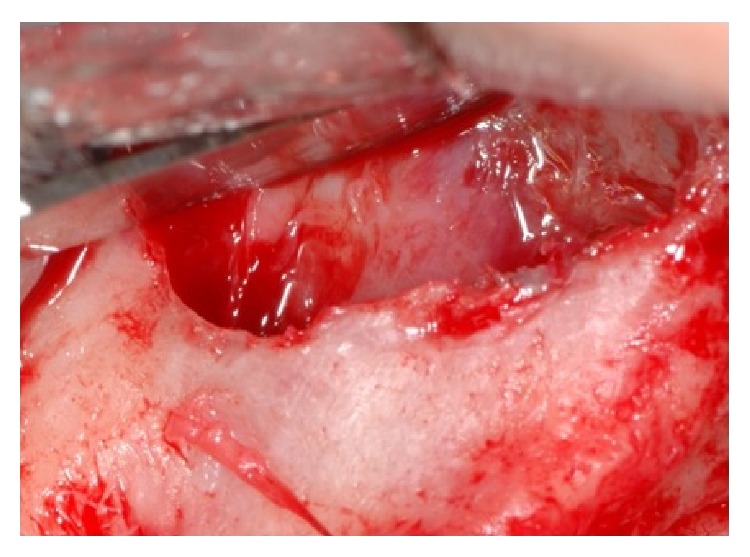
Sinus lift was performed in order to increase the volume of the bone height.

**Figure 5 fig5:**
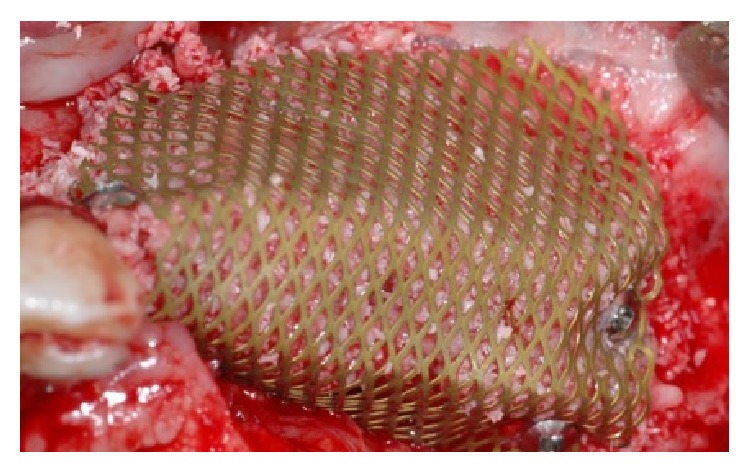
A titanium mesh has been placed for increasing the vertical and horizontal defect.

**Figure 6 fig6:**
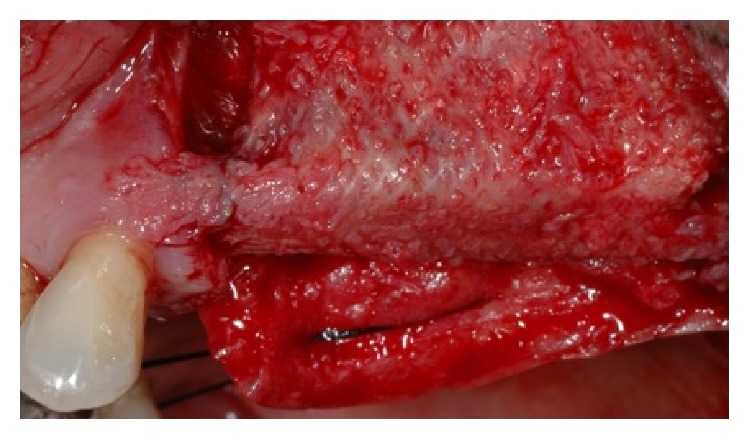
The time titanium mesh removal showed a good healing and the bone volume recovering.

**Figure 7 fig7:**
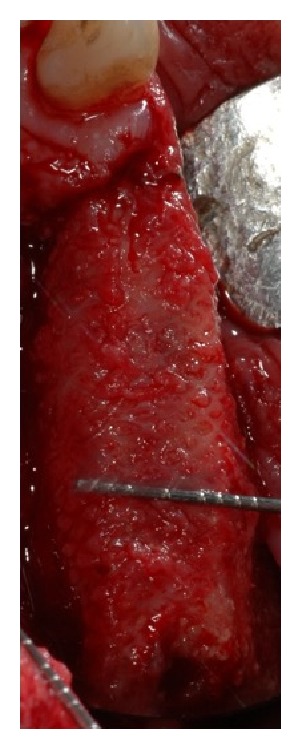
Occlusal view of the increased bone after the bone regeneration procedure with the applied titanium mesh.

**Figure 8 fig8:**
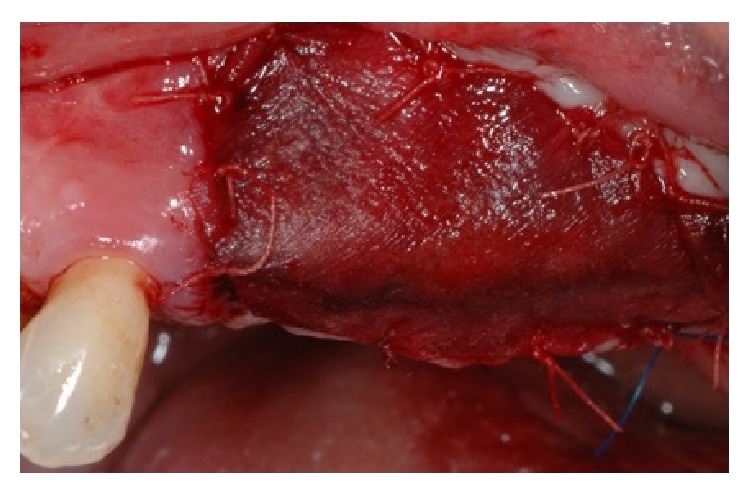
A collagen membrane has been placed in order to favor the soft tissue healing.

**Figure 9 fig9:**
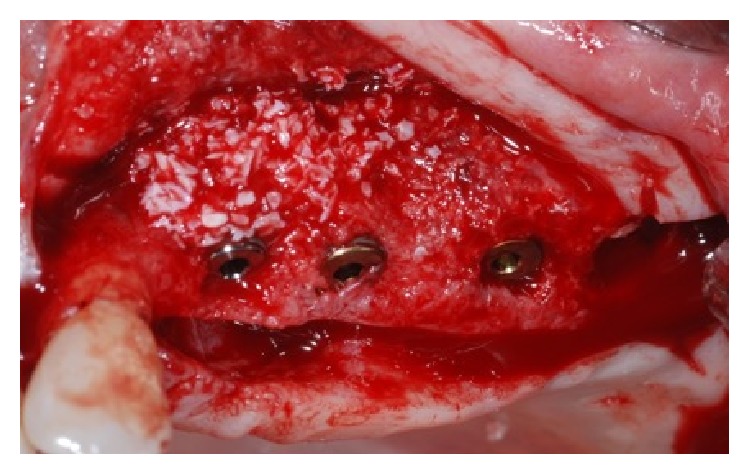
At the time of dental implants the healed bone has been removed and analyzed in order to perform the histologic analysis.

**Figure 10 fig10:**
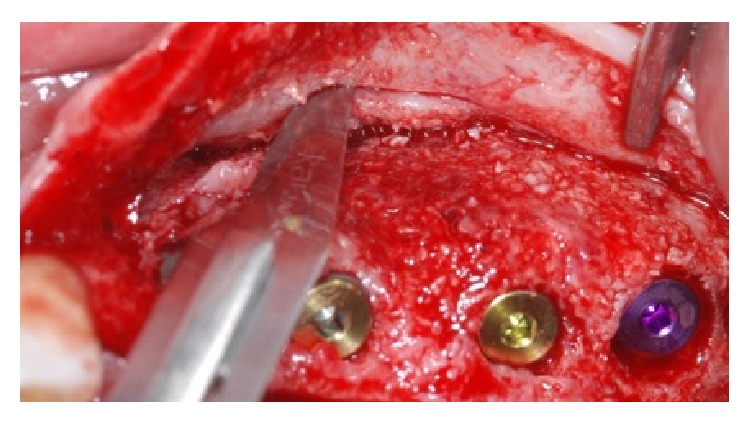
Dental implant was placed and periosteal incision has to be made in order to have a complete soft tissue covering of the dental implants placed.

**Figure 11 fig11:**
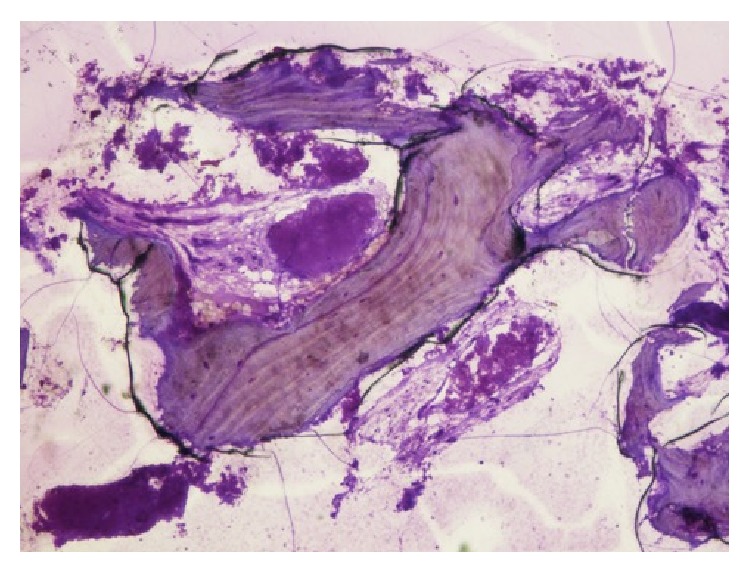
Histological image 40x magnification underlines the presence of new bone cells and some residual particles of the substitute material.

**Figure 12 fig12:**
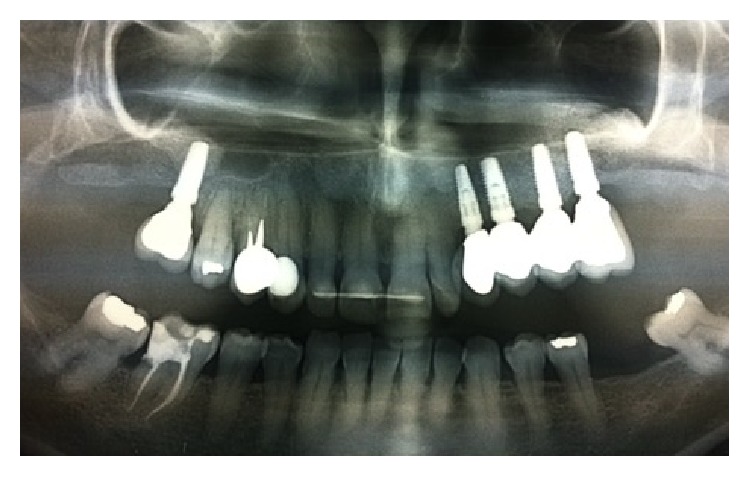
Two-year postoperative X-ray control evidences the integration of the dental implants for support of a fixed prosthetic rehabilitation.

**Figure 13:**
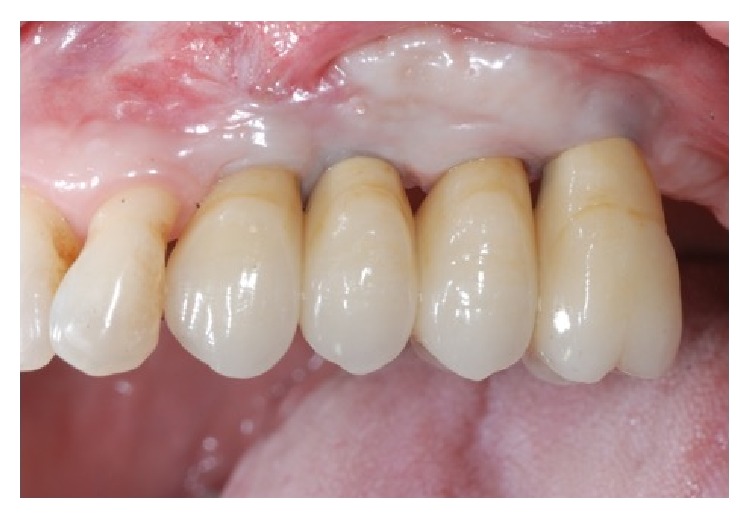
Two-year clinical analyses show a good healing of the soft tissue.

Four months later, implants uncovering and healing abutment connection were done with a simple straight incision at the first right upper molar and a straight incision plus papillae reconstruction according to Palacci [7] technique was performed at the left side. Two months later, impressions were taken, and temporary crowns were placed after two weeks. After five months, impressions for final rehabilitation were taken and a gold ceramic bridge was given. The two-year X-ray orthopantomography showed a physiological contour of the bone levels and the soft tissue appeared clinically stable (Figures 1 [Fig fig2] [Fig fig3] [Fig fig4] [Fig fig5] [Fig fig6] [Fig fig7] [Fig fig8] [Fig fig9] [Fig fig10] [Fig fig11] [Fig fig12]–13).

### 2.1. Histological Analysis

For the augmented area, one cylindrical bone biopsy was taken using a trephine bur with an inner diameter of 2.6 mm. The biopsy was fixed in 10% neutrally buffered formalin for at least 48 h and processed for light microscopy without demineralization by using the Donath and Breuner method [8].

Dehydration was accomplished by increasing ethanol concentrations using a dehydration system with agitation and vacuum. The blocks were embedded in Kulzer Technovit 7200 VLC-resin and sliced longitudinally on an Exakt cutting unit (Exakt, Norderstedt, Germany). Each analyzed slice was reduced by a microgrinding process and then polished using an Exakt grinding unit to an even thickness of 20 mm. These were stained with toluidine blue/pyronine G. Histomorphometric measurements of the tissue fractions (DBBM, autologous bone, newly formed bone and marrow, and/or connective tissue, resp.) were performed only in the augmented area. The sections were digitally photographed using a Leica camera DFC480 fixed on a Leica MZ16 stereomicroscope (Leica, Heerbrugg, Switzerland) using Image Access software (Imagic®, Glattbrugg, Switzerland). The sections were digitally photographed using a Leica camera DFC480 fixed on a Leica MZ16 stereomicroscope (Leica, Heerbrugg, Switzerland) using Image Access software (Imagic, Glattbrugg, Switzerland). Using the same software, the areas were measured by digitally surrounding the contours of the objects. In cases of uncertainty, the areas were compared with the live image using a measured by digitally surrounding the contours of the objects. In this way, the used system is able to clarify the images overlapping the analyzed images with previous acquired data. In cases of uncertainty, the areas were compared with the live image using a Leica DM6000B light microscope at a higher magnification. The results of the histological evaluation underline part from mature cancellous bone, and granules from the Bio-Oss coverage (BO) embedded in newly formed bone are present in the upper part of the biopsy (Figure 11).

## 3. Discussion

A successful implant treatment is determined by taking into account a number of factors: the management of hard and soft tissues, the quality of the prosthetic restoration, and the response to the aesthetic patient's demand.

The combined use of multiple biomaterials and reconstructive techniques could be required in order to obtain the desired result.

The reestablishment of an adequate amount of bone and a proper contour of the alveolar ridge has consequently become mandatory to allow a prosthetically driven implant placement.

Guided bone regeneration has become one of the most proper techniques to achieve this goal. A recent retrospective study on 192 implants placed in augmented bone stated that the cumulative survival rate of the sample was 96%  ±  2% over 6-year mean follow-up period and no statistically significant difference was found between type of graft and membrane [9].

A different and valuable treatment option for maxillary atrophic ridge is related to the use of zygomatic dental implants. Zygomatic implants have been introduced as an alternative to conventional grafting and rehabilitation of severely resorbed maxilla. High success rates and longevity have been documented with zygomatic implant; however, its use is related with a high surgical skill and, at the same time, the prosthodontics final rehabilitation is only a full arch overdenture because a prosthodontics ceramic cemented crown restoration over zygomatic implant is not currently documented [10]. Moreover, the limited evidence of using zygomatic implants in partial rehabilitations with short follow-up times compared to the existing evidence for bone grafts is well documented in the current literature [11, 12].

In the presented clinical case the authors opted for the bone regeneration technique by means of a titanium mesh plus mixed autologous and xenogenic bone mineral particles. The real advantage of this device, as well documented in literature, is the rare risk of superinfection in case of exposure. In a recent systematic review only in 20% of the cases the mesh removal was necessary, while in the remaining cases, it was sufficient to treat the dehiscence with a topical application of chlorhexidine gel, not jeopardizing the final implant rehabilitation despite the less quantity of regenerated bone [13]. A recent work of Lizio et al. found a significant correlation between lack of regenerated bone and time and extent of early exposure [14].

The rationale of mixing autogenous bone with deproteinized bovine bone mineral (DBBM) is to combine the scaffold properties of the xenograft to the osteogenic and osteoinductive properties of the autograft [15, 16].

Moreover, the use of this combination allows for a reduction of the amount of autogenous bone harvested, subsequently decreasing the morbidity related to the technique, the surgical time required to complete the graft, and postoperative discomfort of the patient.

In the presented clinical case, at mesh removal, a connective tissue layer was present in some of the specimens between the mesh and the regenerated bone. This periosteal-like tissue has been described in literature [17] and has been shown particularly evident when titanium meshes are employed for alveolar ridges reconstruction instead of e-PTFE membranes.

Simion et al. [18] conducted an histological and histomorphometrical evaluation of the 1 : 1 mixture of deproteinized bovine bone mineral and autogenous bone graft associated with an expanded-polytetrafluoroethylene (e-PTFE) membrane for vertical ridge augmentation in the human. The histological analysis showed new bone formation and ongoing remodeling of the autogenous bone and the DBBM particles. Both in simultaneous and staged surgical approach, the regenerated bone led to proper osseointegration of dental implants inserted. The authors observed that histologically DBBM underwent very slow resorption and substitution with new bone. Both the autogenous bone and the DBBM particles undergo evident resorption during the healing period from 6 to 9 months. This is demonstrated by the observed mean DBBM density of 8.63% and the autogenous bone density of 6.23% in the 1 : 1 mixture group starting from a hypothetical density of about 25–50% for each material (considering the space between the particle occupied by the blood clot) at the time of the regenerative surgery. Additional evidence regarding the resorbability of DBBM comes from the occasional observation of typical bright beams with adjacent osteoclasts around the particles, indicating active demineralization and remineralization with a physiological remodeling pattern [18].

The peculiarity of DBBM resorption pattern could represent an advantage for the long-term stability of regenerated bone.

One of the problems that the clinician has to deal with, after a regenerative bone procedure, is the deepening of the vestibule and the recreation of the adequate quantity of keratinized mucosa.

As a matter of fact the coronally advanced flap used to obtain a primary wound closure often causes an inadequate vestibular depth at the moment of the second stage surgery. Moreover, the emergence of the implant would appear in the mucosa with aesthetic and functional consequences.

In those cases, a vestibuloplasty is the treatment of choice. This surgical procedure is primarily used to optimize the jaws for prosthesis integration [19].

The secondary aim is to increase the height of the residual alveolar ridge or to generate a sufficient band of keratinized mucosa around teeth or dental implants [20]. In the present clinical case a porcine collagen matrix has been adopted (Mucograft, Geistlich, Wolhusen, Chiasso). The current literature on the role of peri-implant tissue is conclusive to support augmenting peri-implant soft tissues or increasing the width of peri-implant keratinized mucosa [21].

Current studies have demonstrated that sufficient peri-implant keratinized mucosa prevented peri-implant plaque accumulation and buccal soft tissue recessions and therefore reduced the risk of peri-implant mucositis and peri-implantitis [21, 22].

A prospective clinical trial conducted by Schmitt et al. compared the autogenous free gingival grafts with the graft of collagen matrix. In this work authors concluded that the groups showed similar healing with increased peri-implant keratinized mucosa after surgery and therefore are both suitable for the regeneration of the peri-implant keratinized mucosa with a sufficient long-term stability. Moreover, tissue harvesting procedures with Mucograft are less invalid, surgery time can be reduced, and regenerated tissues have a more esthetic appearance [23, 24].

After getting the new vestibule, in this case report an apical reposition flap plus the papillae reconstruction technique was adopted [25–27] in order to obtain a soft tissue architecture similar to natural teeth morphology. The attached masticatory mucosa is displaced buccally, thereby increasing the tissue volume at the buccal side of the implants. The excess buccal tissue allows for a dissection and rotation of pedicles with the purpose of filling the interimplant space with a papilla like soft tissue. In the end, the soft conditioning has been obtained through screw retained temporary resin crowns.

## 4. Conclusions

The case report showed a wide overview of the most advanced techniques adopted in oral surgery to manage hard and soft tissues in order to achieve the prepathologic conditions for a prosthetically driven implant rehabilitation.

The combined use of the autologous bone and biomaterials could represent the treatment of choice for a complete and less invasive treatment.
